# Analysis of mammalian gene batteries reveals both stable ancestral cores and highly dynamic regulatory sequences

**DOI:** 10.1186/gb-2008-9-12-r172

**Published:** 2008-12-16

**Authors:** Laurence Ettwiller, Aidan Budd, François Spitz, Joachim Wittbrodt

**Affiliations:** 1 Developmental Biology Unit, EMBL-Heidelberg, Meyerhofstraße 1, Heidelberg, 69117, Germany; 2 Structural and Computational Biology Unit, EMBL-Heidelberg, Meyerhofstraße 1, Heidelberg, 69117, Germany; 3 Current address: Heidelberg Institute of Zoology, University of Heidelberg, Im Neuenheimer Feld 230, Heidelberg, 69120, Germany; 4 Current address: Institute of Toxicology and Genetics, Forschungszentrum Karlsruhe, Hermann-von-Helmholtz-Platz 1, Karlsruhe, 76021, Germany

## Abstract

**Background:**

Changes in gene regulation are suspected to comprise one of the driving forces for evolution. To address the extent of *cis*-regulatory changes and how they impact on gene regulatory networks across eukaryotes, we systematically analyzed the evolutionary dynamics of target gene batteries controlled by 16 different transcription factors.

**Results:**

We found that gene batteries show variable conservation within vertebrates, with slow and fast evolving modules. Hence, while a key gene battery associated with the cell cycle is conserved throughout metazoans, the POU5F1 (Oct4) and SOX2 batteries in embryonic stem cells show strong conservation within mammals, with the striking exception of rodents. Within the genes composing a given gene battery, we could identify a conserved core that likely reflects the ancestral function of the corresponding transcription factor. Interestingly, we show that the association between a transcription factor and its target genes is conserved even when we exclude conserved sequence similarities of their promoter regions from our analysis. This supports the idea that turnover, either of the transcription factor binding site or its direct neighboring sequence, is a pervasive feature of proximal regulatory sequences.

**Conclusions:**

Our study reveals the dynamics of evolutionary changes within metazoan gene networks, including both the composition of gene batteries and the architecture of target gene promoters. This variation provides the playground required for evolutionary innovation around conserved ancestral core functions.

## Abstract 2

Analysis of the evolutionary dynamics of target gene batteries controlled by 16 different transcription factors reveals stable ancestral cores and highly dynamic regulatory sequences

## Background

Gene function does not just depend on the biochemical and physical properties of gene products, but also on the spatio-temporal expression of these products within the organism. Consequently, evolution does not just proceed through changes of intrinsic properties of the gene product, but also through modification of its expression pattern in time, space and quantity. A growing number of studies have implicated 'regulatory' evolution as an important aspect of inter-species differences, indicating that changes in the elements that control the expression of gene products make a significant contribution to evolutionary divergence and variation (see [1,2] for recent reviews of known *cis*-regulatory mutations and their significance). However, despite this growing awareness of the significance of evolutionary changes of this kind, most studies have focused on the characteristics of individual promoters [3,4], rather than large-scale analyzes. So far, only a few studies of the evolution of *cis*-regulation have focused on the genome-wide level, mostly in yeast [5-7]. In animals, most comparative studies have used expression analysis [8], although some have compared, in a genome-wide manner, binding site location from chromatin immunoprecipitation (ChIP) experiments performed in two species [9,10]. Pairwise comparison of experimental datasets of this kind has provided a good description of the evolutionary changes along a single lineage. However, to incorporate additional lineages, ChIP experiments should ideally be performed in various species using the same cell type. Given the obvious difficulties to run such experiments over multiple species [5], we applied a similar procedure as previously described [5], in our case focusing on animals.

This computational method investigates the extent of gene battery conservation between many species based on the global conservation of binding elements in the homologous sequences of the target gene sets. In this context, we define a 'gene battery' as all genes directly regulated by a transcription factor (TF) as defined by ChIP experiments in the reference species. We also define the 'binding motif' as the sequence recognized by the TF, and the 'binding sites' as being the possible positions on the DNA sequence where the TF binds.

Focusing on over-represented motifs similar to the known TF binding motif, we then evaluated the profile of over-representation of these binding motifs across the homologous sequences of 25 eukaryote species. Significant overrepresentation of the binding motif from the reference species in another species is indicative of a global conservation of the TF gene battery in this other species.

Studying 16 publicly available ChIP datasets over 25 species, we found several batteries conserved throughout the amniote lineage or beyond, for example, E2F1-E2F4 (E2F), which is conserved from *Homo sapiens *to *Caenorhabditis elegans*. Intriguingly, the metazoan E2F gene battery appears to be conserved in yeast even though it is here likely regulated by Mbp1 instead of E2F. In contrast, other batteries have diverged considerably between closely related species, as exemplified by the change in the POU5F1 and SOX2 networks in mouse compared to human in embryonic stem cells. Within a conserved battery, turnover is a pervasive feature of the corresponding TF binding sites, showing that gene batteries can be conserved in the absence of significant sequence conservation in the associated regulatory regions. The rate of turnover appears to be independent from the extent of battery conservation, suggesting that sequence dynamics is not the driving force for battery evolution. However, the position of binding sites relative to the transcription start site (TSS) is usually conserved, indicating constraints shaping the structure of promoter regions.

## Results and discussion

### Considerable variability in degree of conservation of different batteries

We compiled a set of 16 published ChIP datasets based on various human and mouse TFs that play pivotal roles in a wide range of biological processes (Additional data file 1). Using Trawler [11], we *de novo *identified over-represented motifs corresponding to the TF binding motif in the species in which the ChIP was done (the 'reference' species). A total of 16 binding motifs, one per dataset, were identified (Additional data files 2 and 3). Additional over-represented motifs were also considered if they matched known TF binding motifs.

**Figure 1:**
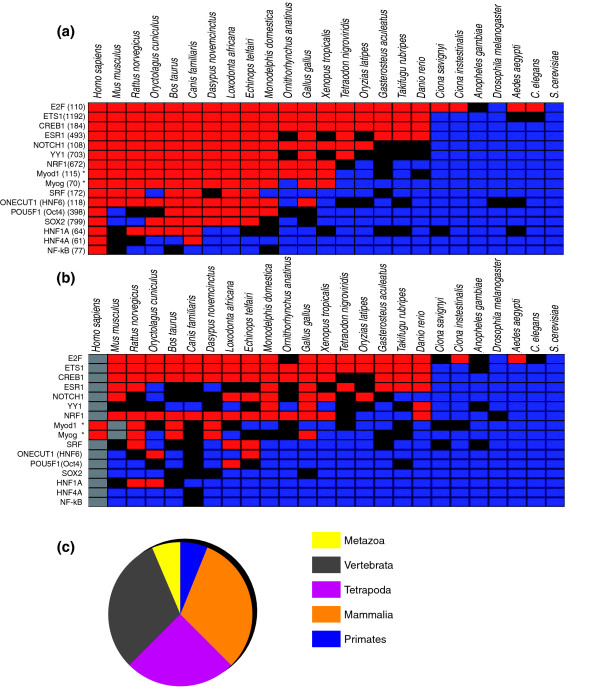
**Conservation of the gene batteries**. **(a) **Conservation profiles of the gene batteries. For each battery, the over-represented motif(s) found in the reference sequence is assessed for over-representation in the corresponding regions of the homologous target genes in 24 other eukaryotic species. The reference species is the one from which the ChIP data were collected (*H. sapiens *or *M. musculus *if labeled with an asterisk). In red are the species whose over-representation score is above 8; in black are the species whose over-representation scores are between 4 and 8; and in blue are the species whose over-representation scores are lower than 4. The higher the over-representation score, the more over-represented is the motif in that species and, hence, the more conserved is the network compared to the reference species network. A significant over-representation score is 4 or above (see Material and methods). The values in parentheses correspond to the number of genes forming the batteries in the reference species. **(b) **Conservation profiles of the regulatory networks using non-alignable sequences: same as (a) except that the sequences used have been masked in the region where a significant alignment can be found with the reference sequence. Grey boxes correspond to the reference species, which, by definition, does not have unaligned sequences. For numerical values, see Additional data file 4. **(c) **Pie chart representing the variable degree of conservation of the various gene batteries analyzed: 1 (6%) gene battery is conserved through the primate lineage (NfKb); 5 (31%) are conserved in most mammals (SRF, POU5F1, SOX2, HNF1A, HNF4A); 4 (25%) are conserved through the tetrapode lineage (Myod1, Myog, NRF1, HNF6 (ONECUT1)); 5 (31%) are conserved through the vertebrate lineage (YY1, ETS, CREB1, ESR1, NOTCH1); and only 1 (E2F) is conserved through the metaozan lineage.

To analyze the dynamics of gene battery evolution, we investigated the presence of these binding motifs in the corresponding homologous regions of 25 eukaryotic organisms, ranging from *H. sapiens *to *Saccharomyces cerevisiae*. Homologous regions are defined by their positions relative to the homologs of the target genes and, hence, do not necessarily align to the reference region. Organisms in which the homologous regions collectively contained a significant over-representation of the reference species' binding motif(s) are identified as having a 'conserved' battery with respect to the reference organism. This is unlikely to be conservation of all the binding sites in all homologs; rather, it is conservation of enough binding sites for us to be able to detect that a statistically significant number of the interactions found in the reference organism are shared by the other organism. We found that global conservation of these batteries are restricted to different sets of organisms for different TFs (Figure 1a and Additional data file 4), corroborating the result previously done in yeast on a different evolutionary scale [5].

While half of the batteries are conserved beyond mammals (Figure 1c), the most ancestrally conserved battery, controlled by E2F [12], is conserved even further into several invertebrates, including *C. elegans*, indicating that a substantial part of the E2F targets have been conserved for at least 990 million years [13]. In the reference species, both the E2F and NF-Y (CBF complex) binding motifs were found to be over-represented. Investigating the evolution of this combination, we found the NF-Y binding motif over-represented in all studied vertebrates, indicating global conservation of the E2F NF-Y combinatorial logic of regulation within the vertebrate lineage (Additional data file 5).

In two cases, SOX2 and POU5F1 [14], we observed strong evidence for a lineage-specific loss of binding motif over-representation in the rodent lineage, most prominently in *Mus musculus *(Figure 1a). This result suggests fundamental differences in the gene regulation by SOX2 and POU5F1, TFs that control pluripotency and self-renewal in human and mouse embryonic stem cells. Such differences have been speculated in previous reports [8,10,15], and our study further shows that these changes are rodent specific. One possible scenario amongst others for such a rodent specific change is the turnover of SOX2 and POU5F1 binding sites into rodent-specific transposable elements, as has been studied previously [15].

### Despite conservation of target genes, many of the predicted binding sites do not align even for closely related species

Regulatory regions are thought to be more conserved than neutrally evolving sequences. To study how the overall conservation of the battery is related to the turnover rate of the binding sites of the corresponding TF, we investigated whether most of the binding sites are located in alignable regions and, thus, have conserved their ancestral locations. To do this, we repeated the same binding motif over-representation analysis using only those regions that could not be aligned with the orthologous region of the reference species (see Material and methods).

In most of the batteries a signal for over-representation of the appropriate binding motif was detected in non-alignable sequences (Figure 1b and Additional data file 4), even for relatively closely related species such as human and mouse (separated by around 75 million years). In more distantly related species the over-representation profiles follow roughly the same pattern as if the entire sequences had been used.

This analysis indicates that many binding sites are found in non-alignable sequence and is consistent with other studies [9,16-19]. This could be due either to the binding sites failing to retain their ancestral positions or to such a high rate of base substitution around the ancestral binding site that it is no longer possible to obtain significant alignments of these regions. In both scenarios, whether change in the binding site or the flanking sequence is responsible, binding sites lose their ancestral genomic context and can, therefore, be considered as turned-over.

Despite wide-spread turnover, we detected a bias in the position of the binding sites relative to the TSSs for most of the gene batteries analyzed (Additional data file 6). This positional bias is conserved in all species where the battery is conserved (Additional data file 11). Taken together, these results indicate that turn-over occurs only within a spatially restricted interval and follows functional constraints (for example, interactions with the basal transcription machinery) that act on the evolution of the promoter architecture.

Next, we investigated whether the turnover-rate is similar for the different batteries. In particular, we investigated whether batteries that are conserved over long evolutionary distances (that is, E2F, CREB1) have a lower rate of turnover due to stronger sequence constraints compared to the batteries that are conserved only within the mammalian lineage. If this were the case, we would expect motif over-representation in non-alignable sequences to be detectable only between more distantly related species for batteries conserved through long evolutionary distances. We found, however, that detection of such over-representation starts at 75 million years independent of the extents of the battery conservation (Figure 1b). This result shows that there is no correlation between the rate of binding site mobility within a regulatory region and the extent of battery conservation. Consistent with this observation, we therefore speculate that turnover of binding sites within the control locus of a gene is mostly the consequence of a genetic drift rather than an active selection.

### A significant number of genes in the gene battery are conserved in most species and form the ancestral core battery

When considering conservation of a gene battery across several species, two evolutionary scenarios can be envisioned: regulatory regions of all genes in the battery are equally likely to retain the binding site(s), hence each gene is equally likely to be lost from the battery; or this probability is highly variable, with certain gene regulatory regions having conserved the binding site(s) in all or most species considered. The latter scenario would argue for the presence of an ancestral regulatory core (those genes for which the probability of loss is particularly low).

**Figure 2:**
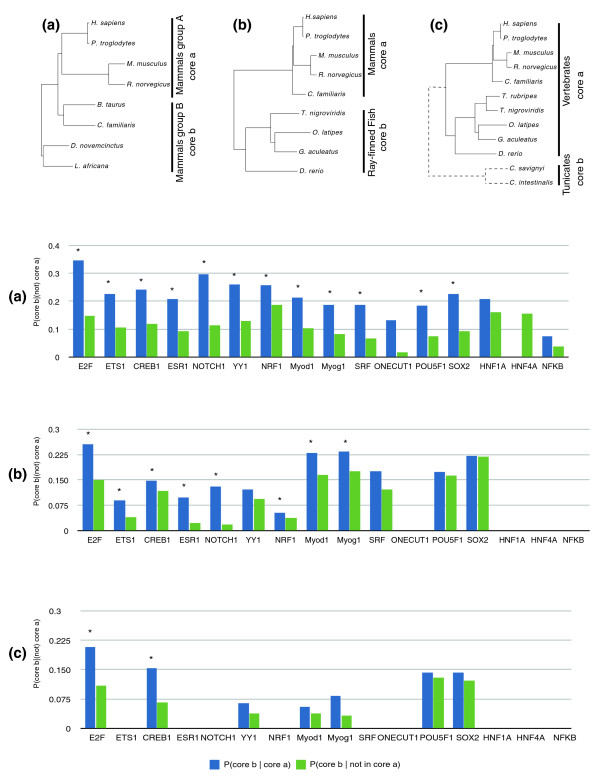
**Assessment of the ancestral cores**. For each gene battery we show the probability of the genes to be part of the ancestral core for lineage b given that the genes are part (blue) or not (green) of the ancestral core of lineage a. Significant differences between P(core b | core a) and P(core b | not in core a) are indicated by asterisks (*p*-values < 0.001). Three phylogenetic distances were considered: **(a) **mammalian; **(b) **vertebrates; **(c) **chordates.

To distinguish between these scenarios, we assessed individual genes in each of the batteries and tested whether the binding motif was found in all or most of the species in a given lineage. To exclude identifying an ancestral core simply by chance, we calculated the probability of a gene being part of an independent lineage core (the lineage not leading to the reference genome) given that the gene is or is not in the ancestral core of the lineage leading to the reference genome. We generated *p*-values using the hypergeometic intersection statistics of the two core sets. The overlap of the ancestral core in the two independent branches forms the ancestral core at the root of the two lineages. In most of the batteries the ancestral core hypothesis is supported at various phylogenetic distances (Figure 2), suggesting that such a core battery represents an invariant network composed of ancestral associated targets indicative of the original function of the corresponding transcriptional regulator (Additional data file 7 and Figure 2).

Compared to other gene batteries, those for E2F and CREB1 have significant ancestral cores over relatively long lineages. These are also the two batteries with the highest overall degree of gene-battery conservation. For E2F, the vertebrate ancestral core contains MCM6 (Additional data file 8), which is essential for the initiation of eukaryotic DNA replication [20,21] by ensuring that DNA replication occurs only once in the cell cycle. We also detected CDC6 as a member of this ancestral network, another essential protein for the initiation of DNA replication. The number of replication initiation genes increases in the vertebrate ancestral core with the presence of genes coding for the polymerase subunits (POLA1 and POLA2). In light of these results and consistent with other findings [22], we speculate that the ancestral role of E2F in the cell cycle is to control replication initiation. Interestingly, two batteries (Myod1 and SRF) contain the trans-regulator gene itself in the vertebrate and mammalian ancestral cores, respectively (Additional data file 8). Thus, feed-back loops were originally present in the ancestral core of these transcriptional regulators and have been well conserved since then.

For a few TFs, promoter ChIP experiments have been performed using two species (human and mouse) [9]. For one TF (E2F [22]) we also found significant cores at various phylogenetic distances using an independent dataset from Ren *et al*. [12]. In order to compare our data with the human-mouse core previously defined experimentally, we divided the experimental set of human E2F bound genes into two categories: genes for which orthologous genes in mouse are bound by E2F (87 genes); and genes for which the mouse orthologs are not bound by E2F (297 genes). The first category can be considered as an ancestral core between human and mouse and, consequently, these genes should overlap with our core datasets. Indeed, we find that a much larger fraction of the human-mouse core overlaps with our ancestral E2F cores at all the phylogenetic distances considered compared to the non-core genes (mammalian, 8% versus 2%; vertebrate, 6% versus 0.6%; and chordate, 2% versus 0.3%), further validating the ancestral core hypothesis.

### Mode of regulatory network evolution

Where the battery is not conserved, several scenarios can explain this lack of conservation. Since we focused our analysis on promoter regions, extensive changes in the localization of the regulatory regions that link the TF to its target genes (from the proximal promoter region to more distal positions) could account for an apparent loss of conservation, but only if such dramatic remodeling of the *cis*-regulatory architecture affected most of the genes involved (a possible scenario for the SOX2 and POU5F1 gene batteries in rodent).

As previously reported in yeast [5], a loss of regulatory network conservation can be caused by a change in the TF controlling that network. This change could be either an alteration of the binding motif recognized by the TF or, more drastically, a cooption of a regulatory system by a different TF. For each of the TFs, we analyzed the conservation of those amino acid residues important for sequence-specific DNA-binding (Additional data file 11). For all TFs analyzed, we identified in most organisms at least one protein expected to bind to the binding motif (Additional data file 9). This indicates that the driving force of gene-battery evolution is mostly in *cis *rather than in *trans*.

Next we investigated replacement of the TF. For this purpose, instead of estimating the enrichment in orthologous sequences of the over-represented binding motif, we applied the *de novo *motif discovery algorithm directly on the orthologous sequence sets. The rationale being that if another motif is found over-represented, it would correspond to the binding motif of the replacement TF. As expected, for most of the batteries no signal was found. For the E2F battery, however, we found that the yeast orthologous sequences contain a different over-represented motif that resembles the E2F motif in its core, but largely differs in the flanking nucleotides (Additional data file 5). This motif corresponds to the binding motif of Mbp1, a DNA binding protein that forms the MBF complex together with Swi6. Mbp1 binds the cell cycle box (consensus ACGCGT [23]) in promoters of genes controlling DNA replication and repair [24]. The MBF complex is thought to be the analogue of the E2F family in the yeast *S. cerevisiae *[25,26].

As E2F also regulates the cell-cycle in the plant kingdom, the most parsimonious explanation is the cooption by the MBF complex of the E2F gene battery in the yeast *S. cerevisiae*. However, despite related cases reported in the literature [5,7], functional replacements of this kind are the exception rather than the rule as the majority of the evolution seems to happen in *cis*. This is expected, given that changes in the *trans*-factor binding specificity would immediately influence the regulation of many genes at the same time, with a potentially bigger phenotypic effect than the gradual change of individual gene expression.

## Conclusion

We have shown that the extent of gene battery stability greatly varies between *trans*-acting factors. We also observed lineage-specific variation in the rate of gene battery evolution, as exemplified by the POU5F1 and SOX2 gene batteries. Investigating binding site turnover, we find it to be a pervasive feature of promoters that appears to be independent of the stability of the gene battery across evolutionary time. We therefore speculate that turnover has little to do with the dynamics of gene battery evolution but rather is a predominantly neutral process. In most of the batteries, we detected a significant ancestral core indicative of the ancestral function of the TF. Taken together, these results highlight yet again that an alignment-centric view is not a suitable perspective for the analysis of regulatory elements. This holds true even when studying highly conserved processes, and perhaps more importantly, even when comparing closely related sequences. Motif composition is a much more accurate measure of non-coding conservation/evolution and can be used across greater evolutionary distances.

## Materials and methods

### ChIP data

Sixteen publicly available promoter ChIP experiments performed on 16 different *trans*-acting factors from *H. sapiens *and *M. musculus *were used. Details of the datasets used have been previously published [11] with further information in Additional data file 1.

### Species analyzed

The species analyzed are the 27 species available in EnsEMBL version 42 [27], unless otherwise stated. A detailed list of species and genome assembly versions used is available in Additional data file 11.

### *De novo *motif discovery

Trawler [11] was used to *de novo *identify over-represented motifs. Sequences were repeat-masked (default repeat masking procedure by EnsEMBL). The following parameters were used: motif from 1 nucleotide to 20 nucleotides long; maximum number of mismatches = 2; minimum occurrences of motif in sample = 10. The sequence length used for the *de novo *analysis of E2F (Additional data file 5) were either 1,000 (vertebrate), 500 or 250 bp (yeast) in order to take into account the variable intergenic size between mammals and yeast. The background was adjusted accordingly. Only the five families with the highest scores were analyzed and motif matching the studied TF binding motif was selected. For E2F, an additional motif corresponding to the NF-Y binding motif was also selected. NF-Y binding sites (CAAT box) are known to be specifically abundant in promoters of genes regulated during G2/M phase [28] and the binding of NF-Y to its site is dynamic through the cell cycle [29].

### Homology assignment and sequence retrieval

For each gene present in the gene batteries analyzed, the homologous genes in the other species listed in EnsEMBL (see 'Species analyzed' section above) were retrieved using EnsEMBL Compara (version 42). Homologous genes annotated as ortholog_one2many, ortholog_one2one, apparent_ortholog_one2one, ortholog_many2many by Compara were used. If multiple orthologous genes were mapped to one gene, all the genes were used for that species. See Additional data file 4 for a complete list of EnsEMBL gene IDs and homologue gene IDs used.

All the sequences used are repeat-masked sequences downloaded from EnsEMBL (version 42). Sequences of 1 kb were used (except for SOX2 and POU5F1, for which 8 kb repeat-masked sequences were used). These sequences correspond to the regions upstream of the annotated start site (of the longest transcript) in EnsEMBL, and define the sample set for each species and battery analyzed.

For the background set a much larger number of genes (2,000) were randomly picked from the reference species (the species used for the ChIP experiment) and the orthologous genes and repeat-masked sequences were retrieved as described above.

### Over-representation assessment

Each binding motif found by Trawler is described by a set of discrete N-mers (Additional data file 3) that can be mapped to the sequences corresponding to either the sample or the appropriate background. The appropriate background is defined as sequences of the same length and coming from the same species as the sample sequences. We did not include other apes as there is insufficient variation in genomic sequences between the apes to distinguish between neutral regions and regions under selection. The number of positions where at least one of the N-mer (or its reverse complement) matches the sequence is calculated in both the sample (*P*_*s*_) and the background (*P*_*b*_). A position is counted only once even if multiple N-mers map to the same position or overlap with the positions of a N-mer already counted. Additionally, all the possible positions in the sample (*N*_*s*_) and the background (*N*_*b*_) are calculated (see equation 1). These correspond to the length of the sequences minus the size of the motif minus one nucleotide:

(1) *N *= *n*(*S*_*seq *_- *S*_*motif *_- 1)

with *N *being all possible positions, *n *being the number of sequences in the sample or the background set, *S*_*seq *_being the sequence length (in base-pairs) and *S*_*motif *_being the motif length (in base-pairs). The over-representation of the binding motifs in the sample sequence compared to the background sequences in different species is assessed by calculating the cumulative distribution function of the hypergeometric distribution using the R statistical application [30]. The density of this distribution is given by equation 2. The upper tail of the distribution is considered.

(2) $$p(P_{x})=\left(\begin{array}{c} P_{b}\\ P_{s} \end{array}\right)\left(\begin{array}{c} N_{b}-P_{b}\\ N_{s}-P_{s} \end{array}\right)/\left(\begin{array}{c} N_{b}\\ N_{s} \end{array}\right)$$

The over-representation score corresponds to the log of the inverse of *p*(*P*_*x*_) and represents the significance of over-representation of the binding motif. The over-representation score is computed if *p*(*P*_*x*_) < 0.5 else 0 is reported (Additional data file 4).

In order to test how significant the conservation score is, a randomization procedure was applied to all sequences analyzed. For this, random gene batteries have been derived for each transcription factor studied with the same number of genes as the real battery. Genes were randomly picked from the set of protein coding genes annotated in the human or mouse (for Myod1 and Myog) EnsEMBL database. The sequences were retrieved and analyzed as described above and the highest over-representation score (computed as equal to 4) corresponds to the lower limit for significant scores in the real data.

We further investigated whether the extent of the conservation is related to the initial size of the gene batteries and we did not find correlation (r = 0.18; Additional data file 11) ruling out sample size effects.

### Positional bias

To mask unspecific positional effects due to nucleotide bias around the TSS [31], we calculated the frequency of distribution of the occurrence of the binding sites relative to the background distribution upstream of the TSS within random loci.

Binding sites are located within 1 kb upstream of the annotated TSS of all the genes in a gene battery (or their orthologs in other species). The same procedure was also applied to a set of 2,000 random genes of the same species analyzed. The TSS of a gene is defined as being the start of the genes as annotated by EnsEMBL (version 42). The upstream region is divided into bins of 100 bp and the number of occurrence found in each bin is counted for both the sample and the background sets. If *Ni *is the total number of nucleotides in bin *i*, *mi *is the number of occurrence of the binding motif found in bin *i*, *b *corresponds to the background sequences, and *s *corresponds to the sample sequences, then the relative frequency of occurrence *F*_*i *_for bin *i *is:

(3) $$F_{i}=\frac{m_{is}}{N_{is}}-\frac{m_{ib}}{N_{ib}}$$

If the number of motifs found *m*_*is *_or *m*_*ib *_> 2 then equation 3 is calculated, else *F*_*i *_= 0.

### Ancestral networks

If the core hypothesis is true, the distribution of binding motif conservation is not uniform and, consequently, genes that are part of this core should have a much higher probability of retaining the binding motif in all the species derived for the last common ancestor of the two selected lineages (that is, be part of the ancestral gene battery).

Patser [32] was used to search the positions of the binding motif (represented as position frequency matrix (Additional data file 8)) in the homologous sequences (see 'Homology assignment and sequence retrieval' section above). Patser was run with the default parameters and -ls 7. In order to account for false negatives due to wrong orthology assignment or badly annotated TSSs, the ancestral core criteria for all the species to have occurrences of the binding motif in the orthologous region was relaxed to most of the species and only the well annotated species were used.

Three evolutionary distances were considered (see Figure 2 for the phylogenetic tree). First was chordates with two independent branches: a) the vertebrate branch with *H. sapiens*, *Pan troglodytes*, *M. musculus*, *Rattus norvegicus*, *Bos taurus*, *Canis familiaris*, *Tetraodon nigroviridis*, *Oryzias latipes*, *Gasterosteus aculeatus*, *Takifugu rubripes *and *Danio rerio*; b) the tunicate branch with *Ciona savignyi *and *Ciona intestinalis*. For a gene to be in core a and b, the binding motif should be found in the upstream sequences of at least nine and two species. respectively.

Second was vertebrates with two independent branches: a) the mammalian branch with *H. sapiens*, *P. troglodytes*, *M. musculus*, *R. norvegicus*, *B. taurus *and *C. familiaris*; b) the teleost branch with *T. nigroviridis*, *O. latipes*, *G. aculeatus*, *T. rubripes *and *D. rerio*. For a gene to be in core a and b, the binding motif should be found in the upstream sequences of at least five and four species, respectively.

Third was mammals with two independent branches: a) the primate/rodent branch with *H. sapiens*, *P. troglodytes*, *M. musculus *and *R. norvegicus*; b) other mammals with *B. taurus*, *C. familiaris*, *Dasypus novemcinctus *and *Loxodonta africana*. For a gene to be in core a and b, the binding motif should be found in the upstream sequences of at least three and three species, respectively.

A list of genes that are both in core a and b for the three phylogenetic distances considered is available in Additional data file 4. For each distance, we calculated: the probability of a gene being part of the independent lineage core (the lineage not leading to the reference genome) given that the gene is in the ancestral core of the lineage leading to the reference genome (P(core b | core a)); and the probability of a gene being part of the independent lineage core (the lineage not leading to the reference genome) given that the gene is not in the ancestral core of the lineage leading to the reference genome (P(core b | not core a)). All the genes analyzed have homolog assignments in species in both linage a and b and have a binding motif in at least one species from lineage a and b. We also calculated how significantly higher is P(core b | core a) compared to P(core b | not core a) by calculating the cumulative distribution function of the hypergeometric distribution using R phyper(w, x, y, z lower.tail = FALSE). With w = number of genes in both cores a and b, x = number of genes conserved in b, y = number of genes with motif in branch a and b - x, and z = number of genes conserved in a. A value below 0.001 is considered significant.

As further controls, we investigated the distribution of binding motif in the reference sequences upstream of the genes contained or not in the core and found a small but significant difference in the distribution (average motif number 1.7 and 2.1 for the genes in the core and not in the core, respectively; KS test *p*-value 1e-14; Additional data file 10). To rule out the circular argument that multiple binding sites in one sequence can artificially create a core, we repeated the same analysis with only the genes with a single binding motif occurrence in the upstream region of the reference species with essentially no change in the significance of the cores (Additional data file 4). We also repeated the same analysis, masking the region of the sequences that align with the reference species and again found that, despite a decrease of the size of the core, these cores (if existing) are significant (data not shown).

### Promoter alignments

For each gene in a battery, the repeat masked sequences were retrieved as described above. The reference sequences were aligned to the ortholgous sequences in a pairwise fashion using Blastz with default parameters [33]. Positions within a significant alignment (score cutoff K above 3,000) were masked in the orthologous sequences.

This procedure was repeated for all the species studied and for all the regulatory networks analyzed. This procedure was also done on the background composed of the 2,000 randomly picked sequences. The same over-representation analysis as described above was performed on these datasets.

## Abbreviations

ChIP: chromatin immunoprecipitation; TF: transcription factor; TSS: transcription start site.

## Authors' contributions

LE designed, conducted and analyzed the experiments. AB designed, conducted and analyzed the TF protein evolution experiments. LE, AB, FS and JW contributed to the manuscript.

## Additional data files

The following additional data are available with the online version of this paper. Additional data file 1 is a summary of the ChIP data used. Additional data files 2 and 3 are the over-represented motifs. Additional data file 4 provides the numerical values from Figure 1a,b as well as the genes analyzed and their orthologues in the 25 species studied. Additional data file 5 is the *de novo *analysis of over-represented motifs in the orthologous regions of the E2F1/E2F4 bound locus in human. Additional data file 6 shows the positional bias of the binding sites relative to the TSS. Additional data file 7 provides a detailed analysis of the ancestral core. Additional data file 8 shows the composition of the ancestral core and lists the position frequency matrices used to find the cores. Additional data file 9 gives the TFs with conserved DNA-base residues. Additional data file 10 shows the distribution of motif number in core and non-core genes. Additional data file 11 includes supplementary notes.

## Supplementary Material

Additional data file 1 Summary of the ChIP data used. Click here for file

Additional data file 2 Over-represented motifs. Click here for file

Additional data file 3 Over-represented motifs. Click here for file

Additional data file 4 Numerical values from Figure 1a,b as well as the genes analyzed and their orthologues in the 25 species studied. Click here for file

Additional data file 5 *De novo *analysis of over-represented motifs in the orthologous regions of the E2F1/E2F4 bound locus in human. Click here for file

Additional data file 6 Positional bias of the binding sites relative to the TSS. Click here for file

Additional data file 7 Detailed analysis of the ancestral core. Click here for file

Additional data file 8 Composition of the ancestral core and the position frequency matrices used to find the cores. Click here for file

Additional data file 9 Transcription factors with conserved DNA-base residues. Click here for file

Additional data file 10 Distribution of motif number in core and non-core genes. Click here for file

Additional data file 11 Supplementary notes. Click here for file
