# Supplementary material for: Analysis of mammalian gene batteries reveals both stable ancestral cores and highly dynamic regulatory sequences
Source: Genome Biol. 2008 Dec 16;9(12):R172. doi: 10.1186/gb-2008-9-12-r172 (PMC2646276; doi:10.1186/gb-2008-9-12-r172)
Supplement: Additional data file 1 — Summary of the ChIP data used. [file gb-2008-9-12-r172-S1.doc]

**Table S1.** *Summary of the ChIP data used*.

Column 1 corresponds to the name of the transcription factor analysed, column 3 corresponds to the species from which the experiment has been done. Column 4 corresponds to the microarray platform technology. The reference of the corresponding papers is recorded in column 5 and the last column corresponds to the biological material used for the ChIP.

| **Transcription factor(s)** | **Role in the cell** | **Species** | **Platform** | **Reference** | **Tissues/growth** |
| --- | --- | --- | --- | --- | --- |
| E2F1 and E2F4 | *Multifunction*  *Cell cycle control.* [34] | *H. sapiens* | 1.5K and Affymetrix DNA microarrays | [12] | Cell culture (WI-38 cells) |
| SOX2 | *Role in early development*  [14] | *H. sapiens* | Agilent array | [14] | Human ES cell |
| POU5F1 (OCT4) | *Role in early development*  [14] | *H. sapiens* | Agilent array | [14] | Human ES cell |
| Myod1 | *Muscle cell specification and differentiation* | *M. musculus* | In house oligo array | [35] | Mouse embryo fibroblast |
| Myog | *Muscle cell specification and differentiation* | *M. musculus* | In house oligo array | [35] | Mouse embryo fibroblast |
| NF-kappa-B  (CREL sub units) | *Multifunction*  *Essential for initiation of the innate immune response.* | *H. sapiens* | PCR product array | [36] | Human U937 cells treated LPS |
| CREB1 | *Multifunction.* | *H. sapiens* | PCR product array | [37] | HEK293T cells |
| TCF1 (HNF1A) | *Required for normal function of liver and pancreatic islets.* | *H. sapiens* | PCR product array | [38] | Hepatocytes |
| HNF4A | *Required for normal function of liver and pancreatic islets.* | *H. sapiens* | PCR product array | [38] | Hepatocytes |
| ONECUT1 (HNF6) | *Required for normal function of liver and pancreatic islets.* | *H. sapiens* | PCR product array | [38] | Hepatocytes |
| NOTCH1 | *Multifunction*  *Lateral inhibition.* | *H. sapiens* | PCR product array | [39] | T-all cell |
| ESR1 (ERa) | *Estrogen receptor* | *H. sapiens* | ChIP-DSL  (Aviva Systems Biology) | [40] | MCF-7 cells |
| ETS1 | *Multifunction.* | *H. sapiens* | Agilent promoter array | [41] | Jurkat cells |
| NRF1 | *mitochondrial biogenesis and cell cycle control and metabolism* (13) | *H. sapiens* | Affymetrix DNA microarrays | [42] | quiescent (G0) T98G cells |
| SRF | *Role in cell proliferation and cell type specific maintenance (*14) | *H. sapiens* | In house oligo array | [43] | Jurkat, T/G HA-VSMC, and Be(2)-C cells |
| YY1 |  | *H. sapiens* |  | [44] |  |

E2F ChIP from [12] (113 human genes with Ensembl entry), CREB1 ChIP from [37] (186 human genes with Ensembl entry, Ave HEK CREB Binding ratio > 6), NOTCH1 ChIP from [39] (109 human genes with Ensembl entry), Myod1 ChIP from [35] (116 mouse genes with Ensembl entry), Myog ChIP from [35] (72 mouse genes with Ensembl entry), HNF1A ChIP from [38] (65 human genes with Ensembl entry), ONECUT1 ChIP from [38] (119 genes with Ensembl entry), HNF4A ChIP from [38] (63 genes with Ensembl entry), SOX2 ChIP from [14] (803 genes with Ensembl entry), POU5F1 ChIP from [14] (399 genes with Ensembl entry), NfkB ChIP from [36] (crel dataset, LPS treatment, 79 genes with Ensembl entry). 1-kilobase (kb) sequences were used (except for SOX2 and POU5F1 where 8 kb sequences were used). These sequences correspond to the regions upstream of the annotated start site (of the longest transcript) in EnsEMBL and defines the sample set for each gene battery analysed.

For HNF1A, the data is derived from HNF_data_v2.xls, downloaded from <http://jura.wi.mit.edu/cgi-bin/young_public/navframe.cgi?s=22&f=downloaddata> with the filters HNF1alpha p-value < 0.001 and HNF1alpha ratio HEP > 2 for Hnf1_HEP in proximal promoter.

For the ESR1, ETS1, NRF1, SRF and YY1 sets, the id and sequence data is derived from the ChIP Metazoan compendium previously published [45].
