# Supplementary material for: Analysis of mammalian gene batteries reveals both stable ancestral cores and highly dynamic regulatory sequences
Source: Genome Biol. 2008 Dec 16;9(12):R172. doi: 10.1186/gb-2008-9-12-r172 (PMC2646276; doi:10.1186/gb-2008-9-12-r172)

Additional datafile 2: **Over-represented motifs.**

PWMs found by Trawler using the ChIP-bound loci of the pulled-down TF analysed.

Boxed motif correspond to the NF-Y binding motif find in addition of the E2F motif. The over-represented motif found for the NOTCH1 dataset is composed of the canonical binding site for NOTCH1-CSL (TGGGA) , followed by a few additional specific nucleotides. The motif resemble the ESR1 canonical motif.

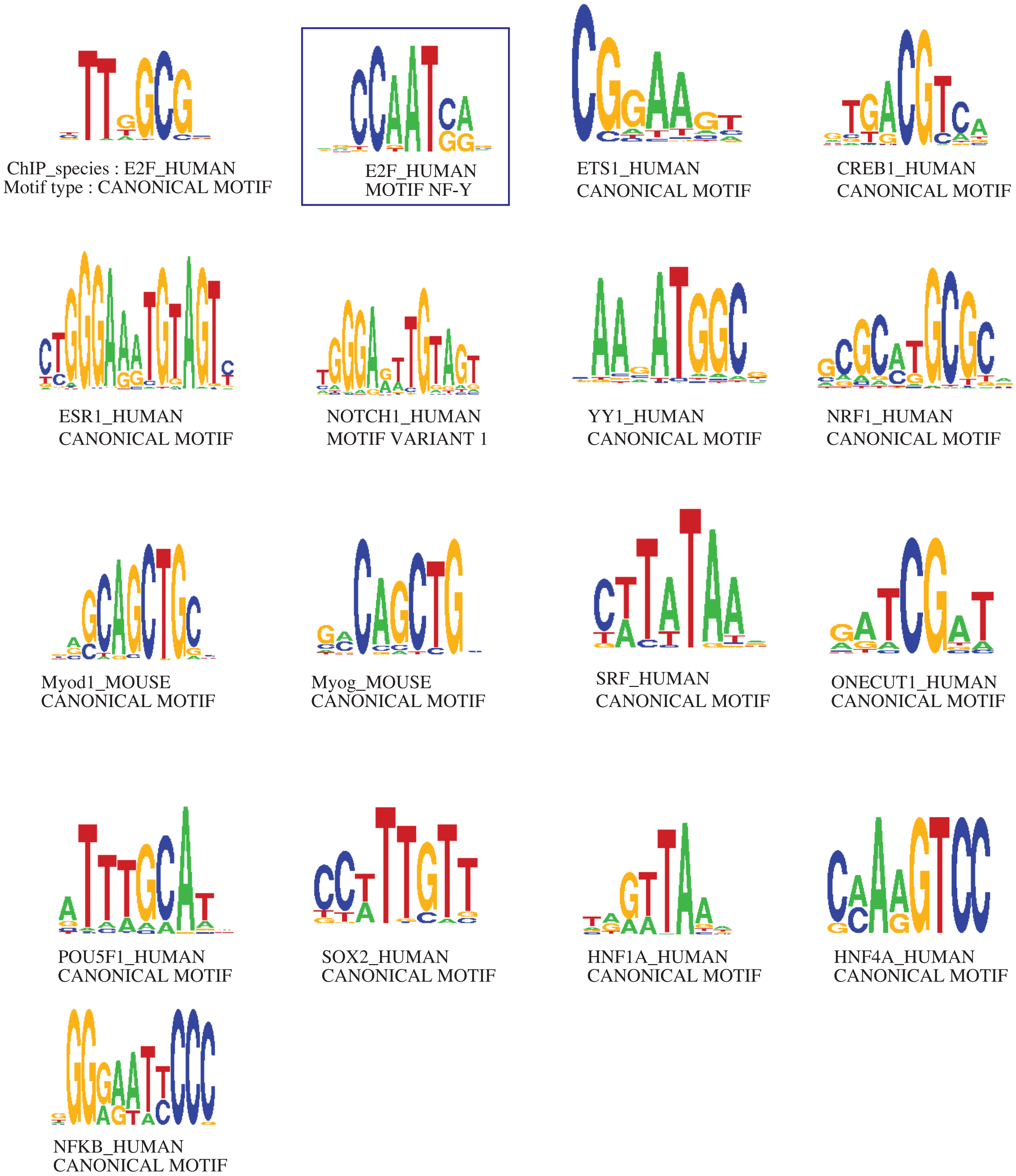

Supplement: Additional data file 2 — Over-represented motifs. [file gb-2008-9-12-r172-S2.pdf]
