# Supplementary material for: Analysis of mammalian gene batteries reveals both stable ancestral cores and highly dynamic regulatory sequences
Source: Genome Biol. 2008 Dec 16;9(12):R172. doi: 10.1186/gb-2008-9-12-r172 (PMC2646276; doi:10.1186/gb-2008-9-12-r172)

Additional data file 5 . **De novo analysis**
De novo analysis of over-represented motifs in the orthologous regions of the E2F1/E2F4 bound locus in human. For each organism, de novo discovered over-represented position weight matrices (PWMs) are reported.

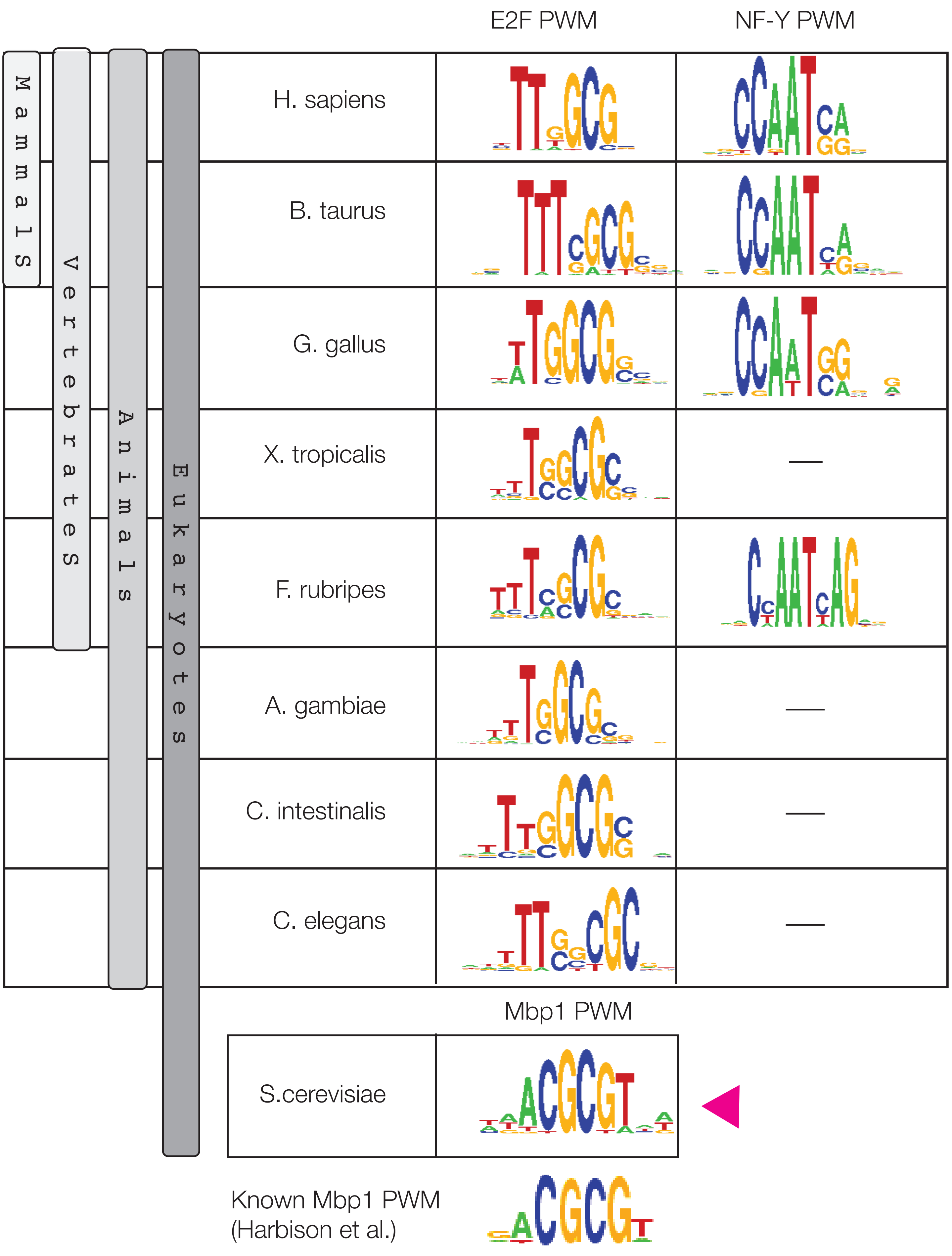

Supplement: Additional data file 5 — De novo analysis of over-represented motifs in the orthologous regions of the E2F1/E2F4 bound locus in human. [file gb-2008-9-12-r172-S5.pdf]
