# Supplementary material for: Analysis of mammalian gene batteries reveals both stable ancestral cores and highly dynamic regulatory sequences
Source: Genome Biol. 2008 Dec 16;9(12):R172. doi: 10.1186/gb-2008-9-12-r172 (PMC2646276; doi:10.1186/gb-2008-9-12-r172)

Additional data file 6. **Positional bias of the binding sites relative to the TSS.**

For each of the gene batteries, the relative frequency of occurrence of the binding motif of the pulled down TF (and the NF-Y motif) is calculated for 1000 base pairs (bp) upstream of the transcription start site (in 100 bp bins). Red bins are positions where collectively, there is more motifs in the set corresponding to the gene battery compare to random sets of genes.

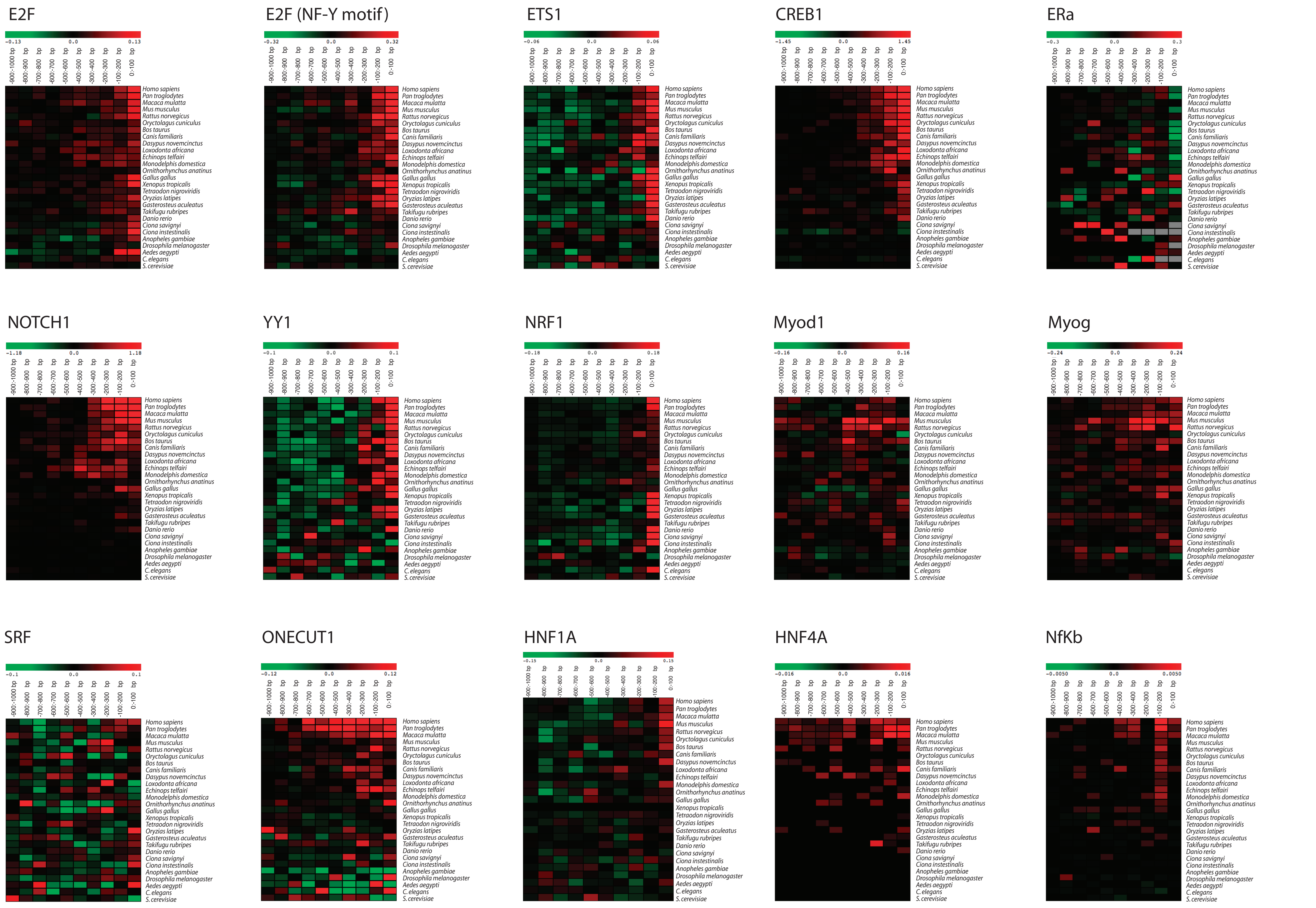

Supplement: Additional data file 6 — Positional bias of the binding sites relative to the TSS. [file gb-2008-9-12-r172-S6.pdf]
