# Supplementary material for: Analysis of mammalian gene batteries reveals both stable ancestral cores and highly dynamic regulatory sequences
Source: Genome Biol. 2008 Dec 16;9(12):R172. doi: 10.1186/gb-2008-9-12-r172 (PMC2646276; doi:10.1186/gb-2008-9-12-r172)

Additional data set 9. **Transcription Factors with Conserved DNA-Base Residues**
Presence of proteins from the same PFAM family as the DNA-binding domains of the transcription factors (TFs) used in ChIP-chip experiments in the proteome of a particular organism is indicated by a filled box. Differently coloured boxes indicate different levels of conservation of residues equivalent to DNA-base binding residues in the transcription factors: red–100% identical; orange–99% to 50% identical; yellow–49% to 0% identical. In the case of NfkB, comparisons are made to the residues in the human NfkB homodimer.

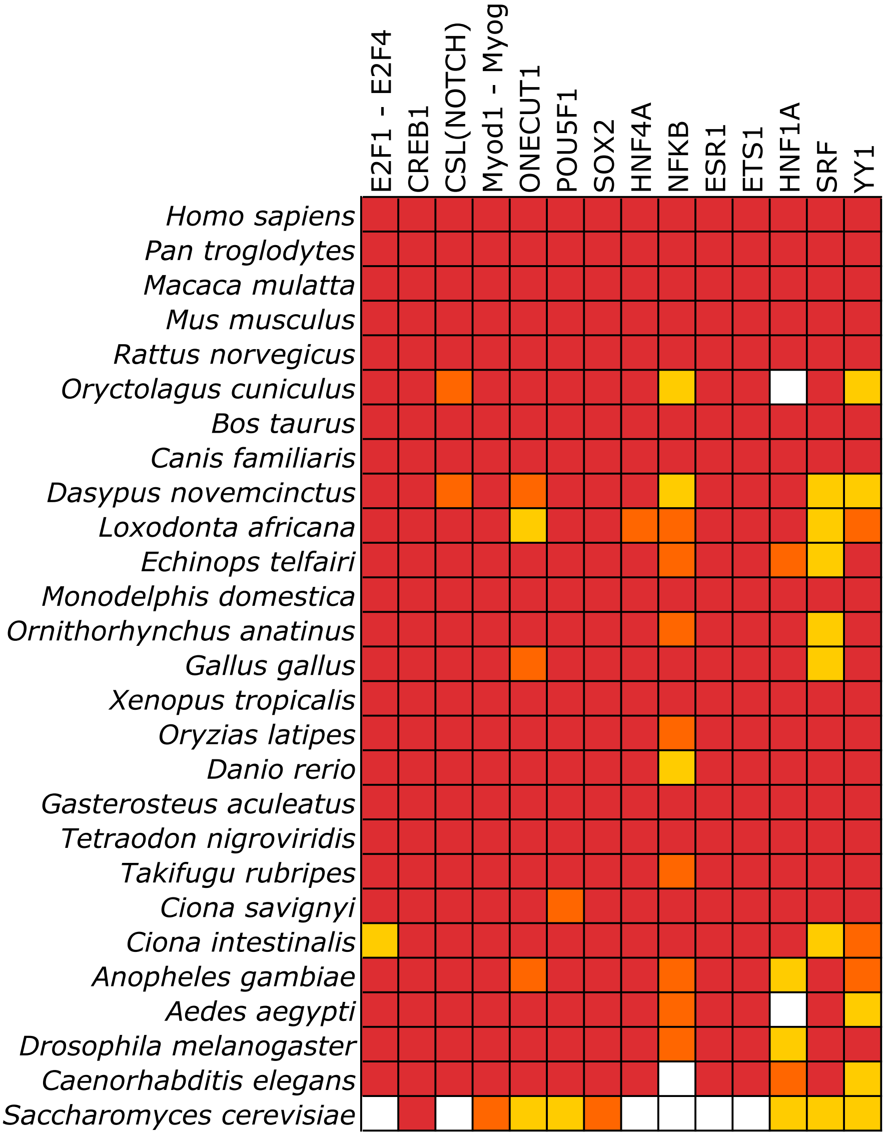

Supplement: Additional data file 9 — Transcription factors with conserved DNA-base residues. [file gb-2008-9-12-r172-S9.pdf]
