# Supplementary material for: Analysis of mammalian gene batteries reveals both stable ancestral cores and highly dynamic regulatory sequences
Source: Genome Biol. 2008 Dec 16;9(12):R172. doi: 10.1186/gb-2008-9-12-r172 (PMC2646276; doi:10.1186/gb-2008-9-12-r172)

Additional data file 10 . **Distribution of motif number in core and non-core genes.**

Distribution of motif number in the reference sequences upstream of the genes contained (red) or not (black) in the core (average motif number 1.7 and 2.1 for the genes in the core and not in the core respectively).

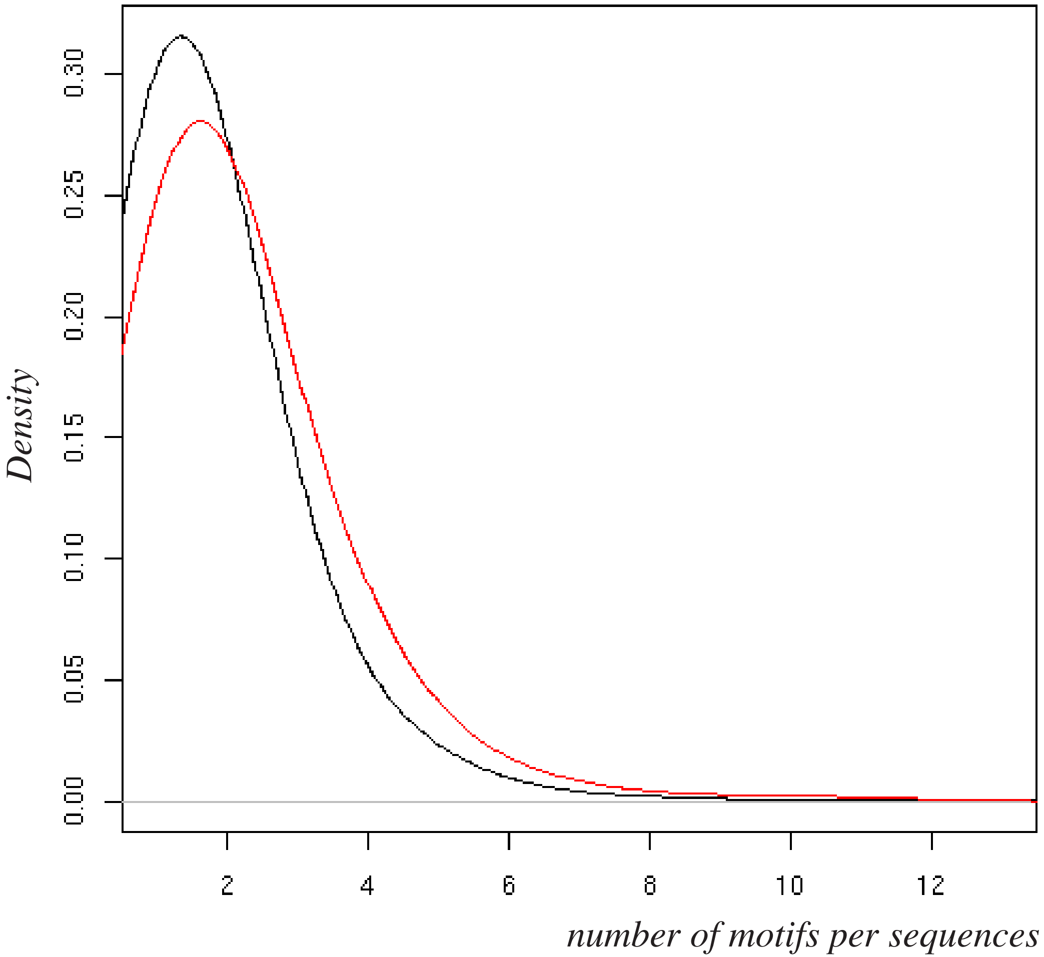

Supplement: Additional data file 10 — Distribution of motif number in core and non-core genes. [file gb-2008-9-12-r172-S10.pdf]
