# Supplementary material for: Analysis of mammalian gene batteries reveals both stable ancestral cores and highly dynamic regulatory sequences
Source: Genome Biol. 2008 Dec 16;9(12):R172. doi: 10.1186/gb-2008-9-12-r172 (PMC2646276; doi:10.1186/gb-2008-9-12-r172)
Supplement: Additional data file 11 — Supplementary notes. [file gb-2008-9-12-r172-S11.doc]

Additional data file 11 : Supplementary notes

## Motif over-representation in the pulled-down loci.

The over-represented motifs from the specific loci bound by the studied TF or the promoter of the TF target genes, were analysed using Trawler [11]. Motifs corresponding to the known binding motif of the pulled-down transcription factor (reference motif) as well as other known binding motif were further selected. For all of the 16 datasets analysed, a motif corresponding to the known binding motif of the TF studied was found in the reference species (the species used for the initial ChIP experiment) **[see Additional data file 2]**. The over-represented motif found for the NOTCH1 dataset [39] is composed of the canonical binding motif for NOTCH1-CSL (TGGGA) [46], followed by a few additional specific nucleotides. These additional nucleotides could be the binding motif of an unknown interacting protein binding very close by. For the E2F ChIP data, the canonical binding motif for NF-Y was also found over-represented and is added to the study **[see Additional data file 2]**. NF-Y binding motif (CAAT box) is known to be specifically abundant in promoters of genes regulated during G2/M phase [28] and the binding of NF-Y to its site is dynamic through the cell cycle [29].

## Correlation between the extend of the gene batteries conservation and the initial size of the sample

To measure the extend of the conservation of the gene battery across animals, a conservation value between 1 to 7 is assigned to a gene battery according to the extend of its conservation (conservation until primate (1), Eutheria (2), Mammals (3), Amniota (4), Tetrapoda (5), Vertebrates (6) and all animal (7)). These values are then correlated with the initial size of the gene batteries and the correlation is calculated using the Pearson's product-moment equation.

| Transcription factor | number of genes | conservation value |
| --- | --- | --- |
| E2F | 110 | 7 |
| ETS1 | 1186 | 6 |
| CREB1 | 184 | 6 |
| ESR1 | 493 | 6 |
| NOTCH1 | 108 | 6 |
| NRF1 | 672 | 5 |
| SRF | 172 | 3 |
| YY1 | 703 | 6 |
| HNF1A | 64 | 3 |
| ONECUT1 (HNF6) | 118 | 4 |
| HNF4A | 61 | 3 |
| POU5F1 (Oct4) | 398 | 3 |
| SOX2 | 799 | 2 |
| Myod | 115 | 5 |
| Myog | 70 | 5 |
| NFkB | 77 | 1 |

we did not find correlation (r = 0.18, see supplementary notes) ruling out sample size effects.

## Conserved positional bias of binding sites relative to the transcription start site (TSS).

We investigated whether other aspects of promoter structure might be conserved, focusing on the distance between binding sites and the TSS. Binding site position bias relative to the TSS has been well documented, notably in yeast [5].

To mask an unspecific positional effect due to nucleotide bias around the TSS [31], we calculated the frequency of distribution of the occurrence of the binding sites relative to the background distribution upstream of the TSS within random loci (see Material and Methods for details).

A bias in the occurrence of the binding sites relative to the TSS can be detected for most of the regulatory networks analysed **[see Additional data file 6]**, with the exception of the POU5F1, SOX2 and SRF networks (data not shown). The distance of these positional biases varies between the different networks, but is generally close to the TSS with the notable exception of the ESR1 binding sites that tend to avoid the immediate 100 bp upstream of the TSS. Binding sites for E2F tend to be very close to the TSS (usually less than 100 bp away) while binding sites for Myod1, Myog and NOTCH1 are distributed further away from the TSS (-300 bp). In most cases, the position bias is also conserved in all species where the regulatory network is conserved. However, we found no significant correlation between genome size and distance of binding sites from TSS.

## Conservation of DNA-Base Binding Residues in Transcription Factors

Identifying Amino Acid Residues Involved in DNA-Base Interactions.

Using BLAST [47] against the PDB [48], we identified structures of proteins either closely related or identical to the TFs used as targets in the various ChIP experiments (in the case of NOTCH1 signaling, we looked for proteins related to CSL). Using the literature, we identified residues in these structures interacting either directly (or via complexed water molecules) with DNA bases (as opposed to sugar-phosphate backbone). In those cases where structures were only available of close relatives of the TFs, the amino acid sequence of proteins whose structure was solved were compared to that used in the ChIP experiment using BL2SEQ [49]. In all cases residues corresponding to DNA-base binding residues in the related sequences were unambiguously identified by these alignments.

DNA-Base-Binding Residue Conservation in Related Proteins

Having identified those regions of the TF from the reference organism targeted by the ChIP experiments (see Identifying Amino Acid Residues Involved in DNA-Base Interactions section), we surveyed other proteomes to analyse the conservation of DNA-base binding amino acids.

Using PFAM [50], we identified the families associated with the DNA-binding function of each of the TFs used as targets (and CSL for the NOTCH pathway) in the relevant ChIP experiments. We then used HMMER 2.3.2 ([http://hmmer.wustl.edu](http://hmmer.wustl.edu/)/, HMMER Software Package) to compare each of these families to the proteomes of the analysed species, collecting all hits to these families with e-value < 0.01. We aligned the sequences of the TFs against each of the proteins containing the same DNA-binding domains using the Smith-Waterman algorithm [51] as implemented by the SSEARCH programme (version 34.26) in the FASTA3 package [52], using settings such that alignments to all of the proteins searched against were included in the output. Using the resulting pairwise alignments, we reported those peptides in each proteome with the greatest identity to the TFs used in the ChIP experiments in those residues involved in DNA-base binding.

In most cases we find proteins whose DNA-base-specific amino acids are identical to those observed in the reference transcription factors (i.e. the majority of cells in supplementary data file 9 are red).

Several organisms, however, are missing such proteins for some of the transcription factors analysed. These organisms tend to be either (a) those with lower-quality genomes (e.g. Oryctolagus cuniculus, Loxodonta africana, and several other mammals, or Ciona intestalis) or (b) relatively distantly related to the reference organism (e.g. Saccharomyces cerevisiae, Aedes aegypti). An obvious explanation for the absence of such proteins in (a) is the lower quality of the predicted proteomes i.e. proteins with identical DNA-base binding amino acids exist in these proteomes, but due to errors and gaps in sequencing/assembly/gene prediction the sequences of these proteins are not present in the predicted proteomes - note that this may also be relevant for some of the more-distantly-related proteomes e.g. that of Aedes aegypti. The more distantly-related an organisms is to the reference organism, the more likely such an absence reflects a true biological difference - this explanation is more likely to be true for case (b) above compared with more closely-related organisms.

## Species and Genome Assembly Version Used for this Study

*Homo sapiens (NCBI 36), Pan troglodytes (PanTro 2.1), Macaca mulatta (MMUL 1.0), Mus musculus (NCBIm36), Rattus norvegicus (RGSC3.4), Oryctolagus cuniculus (RABBIT, May 2005), Bos taurus (Btau 2.0), Canis familiaris (CanFam 2.0), Dasypus novemcinctus (ARMA, May 2005), Loxodonta Africana (BROAD E1, May 2005), Echinops telfairi (TENREC, July 2005), Monodelphis domestica (MonDom 4), Ornithorhynchus anatinus (Oana-5.0), Gallus gallus (WASHUC2), Xenopus tropicalis (JGL4.1), Tetraodon nigroviridis (TETRAODON 7), Oryzias latipes (HdrR Oct 2005), Gasterosteus aculeatus (BROAD S1), Takifugu rubripes (FUGU 4.0), Danio rerio (Zv6), Ciona savignyi (CSAV 2.0), Ciona intestinalis (JGI 2), Anopheles Gambiae (AgamP3), Drosophila melanogaster (BDGP 4.3), Aedes aegypti (AaegL1), Caenorhabditis elegans (WS160), Saccharomyces cerevisiae (SGD1.01).*
